# Test of Butter

**Published:** 1886-01

**Authors:** 


**A Simple Test** of butter, most anybody can apply, is found to be effective as follows : Smear a clean piece of white paper with the alleged buter, roll the paper up and set it on fire. If the butter is pure the smell of the burning paper is rather pleasant, but if the butter is made up wholly or partially of animal fats, the odor is distinctly tallowy.

**Do** not forget that the first of the year, is the best time to subscribe for the JOURNAL OF HEALTH.

